# Major Causes of Death among Older Adults after the Great East Japan Earthquake: A Retrospective Study

**DOI:** 10.3390/ijerph20065058

**Published:** 2023-03-13

**Authors:** Takako Fujimaki, Yuko Ohno, Anna Tsutsui, Yuta Inoue, Ling Zha, Makoto Fujii, Tetsuya Tajima, Satoshi Hattori, Tomotaka Sobue

**Affiliations:** 1 Division of Health Sciences, Graduate School of Medicine, Osaka University, Osaka 565-0871, Japan; 2 Department of Medical Innovation, Osaka University Hospital, Osaka 565-0871, Japan; 3 Division of Environmental Medicine and Population Sciences, Graduate School of Medicine, Osaka University, Osaka 565-0871, Japan; 4 Department of Medical Treatment Recover Care Nursing, Graduate School of Biomedical Sciences, Tokushima University, Tokushima 770-8503, Japan; 5 Department of Integrated Medicine, Graduate School of Medicine, Osaka University, Osaka 565-0871, Japan

**Keywords:** cause of death, death certificate, disaster, earthquake, older adults

## Abstract

This retrospective study investigated the 3-year impact of the Great East Japan Earthquake (GEJE) of 2011 on deaths due to neoplasm, heart disease, stroke, pneumonia, and senility among older adults in the primarily affected prefectures compared with other prefectures, previous investigations having been more limited as regards mortality causes and geographic areas. Using death certificates issued between 2006 and 2015 (*n* = 7,383,253), mortality rates (MRs) and risk ratios (RRs) were calculated using a linear mixed model with the log-transformed MR as the response variable. The model included interactions between the area category and each year of death from 2010 to 2013. The RRs in the interaction significantly increased to 1.13, 1.17, and 1.28 for deaths due to stroke, pneumonia, and senility, respectively, in Miyagi Prefecture in 2011, but did not significantly increase for any of the other areas affected by the GEJE. Moreover, increased RRs were not reported for any of the other years. The risk of death increased in 2011; however, this was only significant for single-year impact. In 2013, decreased RRs of pneumonia in the Miyagi and Iwate prefectures and of senility in Fukushima Prefecture were observed. Overall, we did not find evidence of strong associations between the GEJE and mortality.

## 1. Introduction

The Great East Japan Earthquake (GEJE), which occurred on 11 March 2011, was followed by a large tsunami that struck the northeastern coastal areas of Japan, causing an accident within the Fukushima Daiichi nuclear power plant. Approximately 19,000 people died or are still missing due to the GEJE [1]. In total, over 400,000 survivors were evacuated or stranded a week after the GEJE due to damage to their homes and restricted access to areas surrounding the nuclear power plant [1]. The majority of evacuees were in the Fukushima, Miyagi, and Iwate prefectures, and people aged ≥65 years accounted for over 20% of the evacuees [1]. Vulnerability to disasters is associated with impaired mobility, reduced sensory function, chronic health problems, social isolation, and economic constraints [2,3,4]. In addition, older adults are more likely to have chronic diseases, and the interruption of treatment, stress, lack of food and clean water, extreme heat and cold, and infections due to disaster exacerbate chronic diseases [5,6,7]. In Japan, with its rapidly aging population [8], it is crucial to elucidate the impact of earthquakes on older adults to prepare for the occurrence of disasters.

Several studies have investigated mortality from cardiovascular disease and/or stroke following earthquakes, such as those pertaining to the Northridge Earthquake in 1994 [9], the Great Hanshin Awaji Earthquake (GHAE) in 1995 [10,11,12], the Niigata-Chuetsu Earthquake in 2004 [13], the GEJE in 2011 [14], and both the GHAE and the GEJE [15]. However, many of these studies were limited as regards both causes of death and geographic areas covered. Our study additionally focused on older adults who are especially vulnerable to disasters and at high risk of death.

This study investigated the impact of the GEJE on mortality from five major causes among older adults in the severely affected prefectures compared with other prefectures in Japan. We hypothesized that mortality from the five causes would have increased following the earthquake among older adults in severely affected prefectures compared with those in other prefectures.

## 2. Materials and Methods

### 2.1. Study Design and Data Sources

This retrospective study used population-based death certificate data, which included information on sex, age, and residential address (prefecture). To obtain the relevant statistical data, an application to the Ministry of Health, Labour and Welfare of Japan was required. There were two available population data sources based on the Japanese census data. One estimate by the National Cancer Center of Japan was compiled for the age categories 0–99 years and ≥100 years [16]. Another estimate by the Statistics Bureau of Japan was only compiled for the age categories 0–84 years and ≥85 years [17]. We required population data by the 5-year age categories (0–94 and ≥95 years) for this study. Therefore, we selected population data from the National Cancer Center.

The study was conducted in accordance with the Declaration of Helsinki and approved by the Institutional Review Board of Osaka University (Approval No. 15272).

### 2.2. Participants

**Figure 1:**
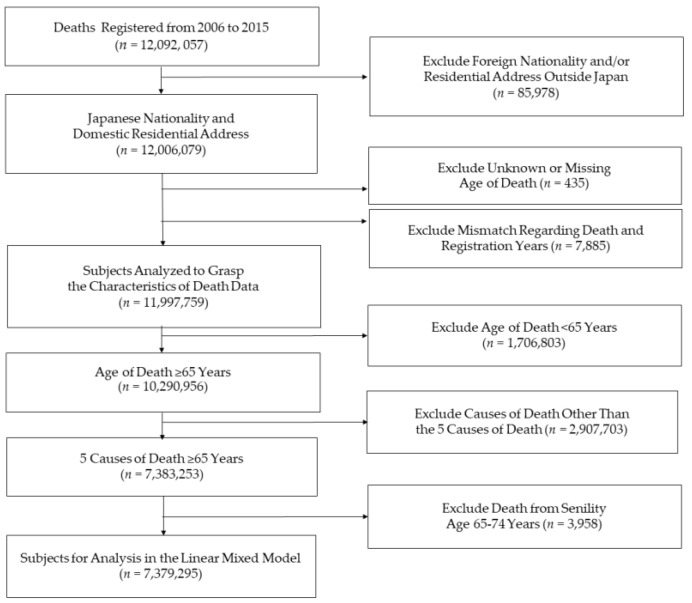
Flow chart of included and excluded participants.

**Figure 2:**
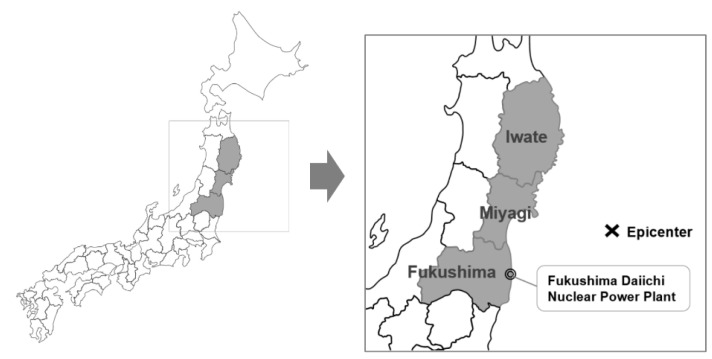
Location of the Fukushima, Miyagi, and Iwate prefectures, which were severely affected by the Great East Japan Earthquake.

Figure 1 shows the flow chart of included and excluded participants. We obtained 12,092,057 death certificates registered between 2006 and 2015. From these data, we excluded those with foreign nationality and/or a residential address outside Japan (*n* = 85,978). Japanese nationals with a domestic residential address accounted for 12,006,079 of these cases. We also excluded those with unknown or missing data for the age of death (*n* = 435) and those with a mismatch between death and registration years (*n* = 7885). A total of 11,997,759 deaths were analyzed. To investigate the mortality rate per 100,000 individuals (MR), we selected adults ≥ 65 years of age (*n* = 10,290,956). For this group, we selected five major causes of death: neoplasm; heart disease, except hypertensive disease; stroke; pneumonia; and senility [18] (*n* = 7,383,253). For deaths due to senility, the ages 65–74 years were excluded from the analysis due to there being a limited number of death registrations or no deaths (*n* = 3958). We analyzed 7,379,295 deaths to investigate the MR. Because the 10th version of the International Statistical Classification of Diseases and Related Health Problems (ICD-10) was applied to classify the primary cause of death in the data, we identified the cause of death using the following ICD-10 codes: C00–97, D00–09, D18.0, D32–33, D35.2–D35.4, and D37–D48 for neoplasm; I01–I02.0, I05–I09, I20–I25, I27, and I30–I52 for heart disease; I60–I69 for stroke; J09–J18 for pneumonia; and R54 for senility. We defined the three prefectures (the Fukushima, Miyagi, and Iwate prefectures) with more than 100 deaths from the GEJE as severely affected prefectures and the remaining 44 prefectures as unaffected prefectures. Figure 2 highlights the location of these severely affected prefectures.

### 2.3. Statistical Analysis

First, we summarized the population and death data from 2006 to 2015. Second, we obtained the MR for each sex, age category (65–69, 70–74, 75–79, 80–84, 85–89, 90–94, and ≥95 years), and prefecture to observe annual trends in mortality. The MR for each group was calculated by dividing the number of deaths by the population number and multiplying by 100,000. We excluded data with zero MR. The subgroup with zero MR was the senility group and was only 0.2%. Third, we used linear mixed models (LMMs) to estimate risk ratios (RRs) and 95% confidence intervals (CIs) in the cause of death in affected prefectures for several single years after the GEJE. The response variable was the log-transformed MR. Adjustment for multiplicity was not performed. The model included the following fixed effects: area category—Fukushima, Miyagi, Iwate, and the remaining 44 prefectures; year of death—each year from 2006 to 2015; age category—65–69, 70–74, 75–79, 80–84, 85–89, 90–94, and ≥95 years; sex—male and female; and interaction terms between the area category and each year of death from 2010 to 2013. In addition, a random effect of intercept for all 47 prefectures in Japan was included. Of the observed years, 2006–2015, we focused on the results for the years 2010–2013. The year of death 2010 was included as a year with no impact from the GEJE. The years of death 2011–2013 were selected to observe RRs after the GEJE, as most of the previous studies investigating long-term deaths after earthquakes had not determined any impact beyond 3 years.

A *p*-value < 0.05 was considered statistically significant. Statistical analysis was performed using SAS 9.4^®^ (SAS Institute Inc., Cary, NC, USA), and the MIXED procedure of SAS was used to perform LMM analyses.

## 3. Results

The characteristics of the population data are summarized in Table A1. The percentages of the mean annual population aged ≥65 years were 25.6%, 22.9%, 27.7%, and 23.6% in the Fukushima, Miyagi, Iwate, and the other 44 prefectures, respectively. The percentage of males aged ≥65 years (40.7–42.7%) was lower than that of females in all area categories.

Table 1 summarizes the characteristics of the death data by age category, cause of death, and sex. The mean annual mortality rates (AMRs) for all causes of death in people aged 0–64 years were 202, 198, 237, and 183 deaths per 100,000 population in Fukushima, Miyagi, Iwate, and the other 44 prefectures, respectively. The mean AMRs for those aged ≥65 years were 3862, 3610, 3836, and 3575 deaths per 100,000 population in Fukushima, Miyagi, Iwate, and the other 44 prefectures, respectively. The older the age category, the higher its AMRs. The area category with the highest mean AMR was Fukushima Prefecture for deaths due to neoplasm, heart disease, and senility, Iwate Prefecture for stroke, and the other 44 prefectures for pneumonia. A comparison of males and females aged ≥65 years showed that AMRs were higher in males for all causes of death.

**Figure 3:**
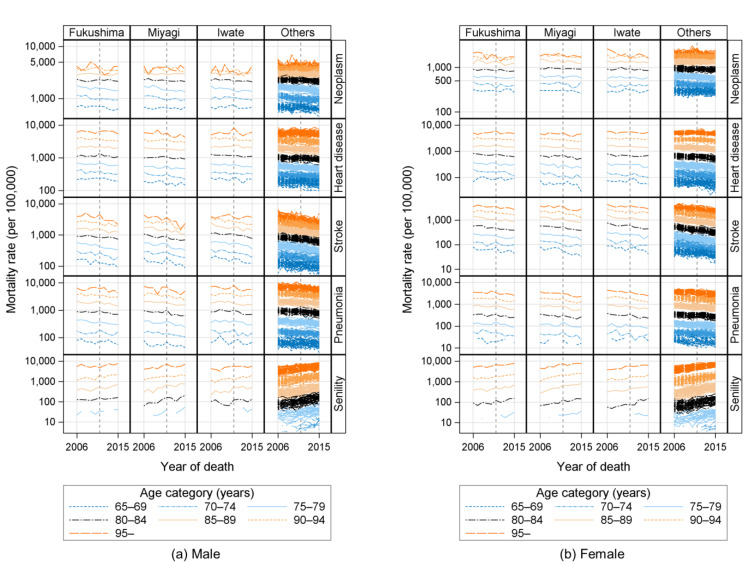
Mortality rates for males and females from 2006 to 2015. The area category Others indicates results for each of the 44 prefectures. Number of deaths by sex, cause of death, year, and prefecture < 10 were excluded from the graph for privacy protection reasons.

Figure 3 shows annual trends in the MRs by cause of death and prefecture for males and females, respectively. These graphs also illustrate the major annual trends for the other unaffected prefectures (*n* = 44). The MRs by cause of death were highest for neoplasm, followed by heart disease, stroke or pneumonia, and senility. The annual trends increased for deaths due to senility and decreased for deaths due to other causes. The MRs were higher in males for all causes of death, and there were large sex differences for deaths due to neoplasm, stroke, and pneumonia. The MRs were generally higher among the older age categories than younger age categories across all causes of death. In a comparison between affected and unaffected prefectures, the MRs due to several causes of death were higher in the affected prefectures than in other prefectures in 2011; however, the results for the sub-age categories differed by age category.

Table 2 summarizes the highlights of the RRs and 95% CIs for fixed effects and interaction terms by cause of death. Table A2 lists the RRs for all the fixed effects and interaction terms. Considering the interaction terms between the area category and the year of death, the RRs for neoplasm showed minor variations from 2010 to 2013, ranging between 0.95 and 1.01 in the three affected prefectures. The RRs for heart disease in the three affected prefectures were slightly lower than those in the 44 prefectures in 2010 (0.99 [95% CI, 0.88–1.12], 0.97 [0.86–1.09], and 0.96 [0.85–1.08] for the Fukushima, Miyagi, and Iwate prefectures, respectively), but were higher than those in the 44 prefectures in 2011 (1.06 [95% CI, 0.95–1.20], 1.03 [0.91–1.15], and 1.06 [0.95–1.20], respectively). The RRs for stroke between 2010 and 2013 were the highest in 2011 in the Fukushima, Miyagi, and Iwate prefectures (1.08 [95% CI, 0.96–1.21], 1.13 [1.01–1.27], and 1.07 [0.96–1.20], respectively). Of these, the RR was significantly higher only in Miyagi Prefecture. The RRs for pneumonia between 2010 and 2013 were higher in the Fukushima, Miyagi, and Iwate prefectures than in the 44 prefectures in 2011 (1.10 [95% CI, 0.99–1.23], 1.17 [1.04–1.31], and 1.04 [0.93–1.17], respectively), although the RR was significant only in Miyagi Prefecture in that year. The RRs were significantly lower in the Miyagi and Iwate prefectures in 2013 (0.86 [95% CI, 0.77–0.97 and 0.88 [0.78–0.98], respectively). In 2011, the RR for senility was significantly higher only in Miyagi Prefecture (1.28 [95% CI, 1.07–1.54]), and slightly lower in the Fukushima and Iwate prefectures (0.98 [95% CI, 0.81–1.17 and 0.97 [0.81–1.16], respectively) compared to the 44 prefectures. For fixed effects, the RR for stroke was significantly high only in Iwate Prefecture (1.37 [95% CI, 1.07–1.74]).

## 4. Discussion

We investigated the impact of the GEJE on mortality in older adults across three severely affected prefectures and compared it to the 44 unaffected prefectures in Japan using the enumerated data. Because no apparent changes were observed in the graphs of MRs post-GEJE (Figure 3), statistical analysis was performed using an LMM. As a result, we found that there were significant increases in the RRs for stroke, pneumonia, and senility in Miyagi Prefecture in 2011, while there were no significant increases in the other areas.

Although we presented the results based on an LMM, we also applied Poisson and negative binomial mixed models. However, these models failed to converge on several causes of death and could not provide estimates. When obtaining estimates, these models produced estimates very similar to those provided by the LMM; however, the LMM yielded more conservative results. Therefore, we have presented our results using the LMM in a unified manner for all causes of death to describe the changes in area-specific MRs during the years shown in Figure 3.

This study showed that the fixed effect for the RR for deaths due to stroke was significantly higher in Iwate Prefecture. However, the RR in the interaction terms between the area category and each year from 2010 to 2013 for deaths due to stroke significantly increased only in Miyagi Prefecture in 2011, and not in Iwate Prefecture. Several previous studies have shown post-earthquake increases in the incidence of death due to stroke [11,15] and the incidence of stroke [19,20,21]. Meanwhile, previous studies have noted no significant increases in the incidence of death caused by stroke [9] and the incidence of stroke [22]. Thus, the impacts of earthquakes on the incidence of stroke were inconsistent. This inconsistency may be related to a variety of factors. Reasons for differences in the impact on the RR among affected prefectures could not be clarified. Hypertension is a known risk factor for stroke [23,24]. Elevated blood pressure after disasters is referred to as disaster hypertension, which has been shown to affect individuals after earthquakes [25,26,27,28,29,30,31,32,33]. One previous study reported that the mean blood pressure increased in both evacuees and non-evacuees after the GEJE, and the change was more pronounced in evacuees compared to that in non-evacuees [33]. Several studies showed that an elevation in blood pressure peaked 1–2 weeks after the earthquake [25,27,31] and that it required 3–6 weeks to recover to the same level of blood pressure as before the earthquake [25,27,28]. These studies suggest that it is important for residents, especially evacuees, to undergo screening from the early phases after the earthquakes to detect elevated blood pressure and subsequently ensure appropriate blood-pressure control.

In the RRs for mortality from heart disease, there was no significant change across all interaction terms. One previous study reported that there was no long-term impact on cardiovascular death, hospitalization for heart failure, and acute myocardial infarction for the three-year follow-up after the GEJE [14]. As regards short-term impact within 15 weeks after the earthquake, there was no significant increase in the incidence of acute myocardial infarction and takotsubo cardiomyopathy [34]. However, numerous previous studies have reported an increase in the incidence of death due to coronary heart disease [9,10,11,12,13,15] and the incidence of coronary heart disease [21,35,36,37,38,39], heart failure [21,34,40,41], out-of-hospital cardiac arrest [21,42,43], and takotsubo cardiomyopathy [44] after devastating earthquakes. Risk factors have been identified for heart disease although many of these factors were not considered in this study. Therefore, future studies should consider these risk factors.

The RRs for death due to pneumonia significantly increased in Miyagi Prefecture in 2011; meanwhile, they decreased in Miyagi Prefecture and Iwate Prefecture in 2013. The reason behind this decrease could not be identified on the basis of previous studies. Several previous studies observed an increase in the incidence of pneumonia in areas severely affected by the GEJE [21,45,46]. Several studies reported that more patients with pneumonia were living in shelters following the GHAE [47] and the GEJE [48]. The number of evacuees after the GEJE was the highest in Miyagi Prefecture, followed by the Fukushima and Iwate prefectures [1]. The importance of proper living conditions, hygiene, and nutrition in shelters has been recommended in the Guidelines for the Management and Prevention of Cardiovascular Diseases in Disasters published after the GEJE [49].

The RRs for death due to senility significantly increased only in Miyagi Prefecture in 2011 and significantly decreased in Fukushima Prefecture in 2013. No previous studies mention a direct impact on deaths due to senility after earthquakes. One previous study reported that physical activity and muscle strength decreased in older adults living in temporary housing compared with those living at home following the GEJE [50]. Another study reported that damage to housing was significantly associated with cognitive decline among older survivors [51]. Meanwhile, factors contributing to the decrease in 2013 remain unclarified.

There was a small variation and no significant increase in the RRs for mortality from neoplasm. Although many medical facilities were affected by the GEJE, it was unknown exactly how many patients with cancer consequently experienced a delayed diagnosis or interruption in treatment. A study reported that hospitalization for the progression of lung cancer increased slightly during the first 2 months following the GEJE, but not significantly [45].

This study had several limitations. First, the data did not include information on other important influencing factors on death, such as comorbidities, daily activity levels, and whether the participants were evacuated after the GEJE. Second, a comparison between the seriously damaged coastal and the less-damaged inland areas in each prefecture was not included in the data. Lastly, the interpretation of *p*-values should be exploratory only, since the estimated *p*-values were not adjusted for multiple tests. Therefore, future studies are required to assess the association between the cause of death following a disaster and individual background characteristics.

## 5. Conclusions

We observed increases in the RRs of death due to stroke, pneumonia, and senility in the Miyagi Prefecture in 2011 among older adults, whereas decreases in the RRs of death due to pneumonia n the Miyagi and Iwate prefectures and senility in Fukushima Prefecture in 2013. The reason for this decrease could not be identified in previous studies. No strong association was observed between the GEJE and mortality; however, our results suggest this would have been significant only for the single-year impact. Future studies are warranted to assess the association between the cause of death following a disaster and individual background characteristics.

## Figures and Tables

**Table 1:** Characteristics of the death data from 2006 to 2015.

	Fukushima Pref.	Miyagi Pref.	Iwate Pref.	Others
	Mean	Mean	Mean	Mean
	AMR ^a^ (*n* ^b^)	AMR ^a^ (*n* ^b^)	AMR ^a^ (*n* ^b^)	AMR ^a^ (*n* ^b^)
All death	1140 (22,860)	978 (22,775)	1238 (16,266)	1045 (25,861)
Age category				
0–64 years	202 (3026)	198 (3565)	237 (2259)	183 (3678)
≥ 65 years	3862 (19,834)	3610 (19,210)	3836 (14,007)	3575 (22,183)
65–69 years	1071 (1328)	1014 (1404)	1117 (974)	973 (1782)
70–74 years	1666 (1921)	1631 (2033)	1679 (1424)	1553 (2453)
75–79 years	2804 (3018)	2766 (3042)	2792 (2196)	2677 (3465)
80–84 years	4882 (4253)	4860 (4079)	5036 (3041)	4744 (4485)
85–89 years	8705 (4489)	8744 (4.06)	8789 (3026)	8375 (4570)
90–94 years	15,284 (3180)	15,172 (2.09)	15,340 (2169)	14,714 (3428)
≥95 years	26,614 (1645)	25,985 (1.37)	26,166 (1176)	25,715 (2000)
				
Causes of death (age ≥ 65 years)				
Neoplasm	1006 (5160)	994 (5285)	987 (3605)	992 (6366)
Heart disease	699 (3590)	566 (3008)	668 (2441)	589 (3628)
Stroke	477 (2443)	440 (2327)	531 (1934)	388 (2324)
Pneumonia	400 (2051)	352 (1864)	396 (1442)	405 (2479)
Senility	222 (1151)	213 (1158)	187 (688)	185 (1116)
Total of five causes	2805 (14394)	2565 (13,642)	2769 (10110)	2559 (15,913)
				
Sex (age ≥ 65 years)				
Male	4595 (9793)	4244 (9532)	4645 (6894)	4206 (11,125)
Female	3343 (10,041)	3146 (9678)	3282 (7113)	3121 (11,057)

^a^ Annual mortality rate (AMR) was calculated by dividing the sum of the annual mortality rate per 100,000 population between 2006 and 2015 by 10 years. In addition, the mean values for the area category “Others” were divided by 44 prefectures. ^b^ Mean number of deaths was calculated by dividing the sum of the mean number of deaths between 2006 and 2015 by 10 years. In addition, the mean values for the area category Others were divided by 44 prefectures. Pref.: Prefecture; Others: the 44 prefectures other than the Fukushima, Miyagi, and Iwate prefectures; five causes of death: neoplasm, heart disease, stroke, pneumonia, and senility.

**Table 2:** Risk ratios and confidence intervals for the fixed effect and interaction terms between the area category and the year of death.

	Neoplasm		Heart Disease		Stroke		Pneumonia		Senility	
Parameters	RR (95% CI)	*p*-Value	RR (95% CI)	*p*-Value	RR (95% CI)	*p*-Value	RR (95% CI)	*p*-Value	RR (95% CI)	*p*-Value
**Area category (Fixed effect)**										
44 prefectures	Ref.		Ref.		Ref.		Ref.		Ref.	
Fukushima Pref.	1.00 (0.90–1.11)	0.979	1.17 (0.96–1.43)	0.128	1.20 (0.95–1.53)	0.134	0.96 (0.77–1.20)	0.731	1.35 (0.79–2.32)	0.278
Miyagi Pref.	1.01 (0.91–1.12)	0.882	0.99 (0.81–1.22)	0.956	1.16 (0.91–1.48)	0.221	0.89 (0.71–1.12)	0.319	1.26 (0.73–2.16)	0.407
Iwate Pref.	0.99 (0.89–1.10)	0.825	1.12 (0.92–1.37)	0.259	1.37 (1.07–1.74)	0.011 ^†^	1.00 (0.80–1.26)	0.965	1.06 (0.61–1.81)	0.846
										
**Interaction terms**										
44 prefectures * Year 2010	Ref.		Ref.		Ref.		Ref.		Ref.	
Fukushima Pref. * Year 2010	1.01 (0.95–1.07)	0.730	0.99 (0.88–1.12)	0.895	1.04 (0.92–1.16)	0.546	1.03 (0.92–1.15)	0.589	0.89 (0.74–1.07)	0.217
Miyagi Pref. * Year 2010	1.01 (0.96–1.07)	0.643	0.97 (0.86–1.09)	0.624	1.08 (0.96–1.21)	0.179	0.93 (0.84–1.05)	0.238	1.16 (0.96–1.39)	0.117
Iwate Pref. * Year 2010	0.99 (0.93–1.05)	0.689	0.96 (0.85–1.08)	0.482	0.99 (0.88–1.11)	0.850	1.02 (0.91–1.14)	0.741	1.02 (0.85–1.23)	0.816
44 prefectures * Year 2011	Ref.		Ref.		Ref.		Ref.		Ref.	
Fukushima Pref. * Year 2011	0.98 (0.92–1.03)	0.435	1.06 (0.95–1.20)	0.302	1.08 (0.96–1.21)	0.200	1.10 (0.99–1.23)	0.086	0.98 (0.81–1.17)	0.789
Miyagi Pref. * Year 2011	0.97 (0.92–1.03)	0.334	1.03 (0.91–1.15)	0.674	1.13 (1.01–1.27)	0.031 ^†^	1.17 (1.04–1.31)	0.007 ^†^	1.28 (1.07–1.54)	0.007 ^†^
Iwate Pref. * Year 2011	0.99 (0.94–1.05)	0.719	1.06 (0.95–1.20)	0.294	1.07 (0.96–1.20)	0.224	1.04 (0.93–1.17)	0.480	0.97 (0.81–1.16)	0.733
44 prefectures * Year 2012	Ref.		Ref.		Ref.		Ref.		Ref.	
Fukushima Pref. * Year 2012	0.96 (0.91–1.01)	0.140	1.01 (0.90–1.14)	0.807	0.95 (0.84–1.06)	0.345	1.04 (0.93–1.16)	0.532	0.85 (0.71–1.02)	0.075
Miyagi Pref. * Year 2012	0.99 (0.94–1.05)	0.752	0.94 (0.84–1.06)	0.304	0.97 (0.86–1.09)	0.577	0.98 (0.87–1.09)	0.686	0.92 (0.77–1.11)	0.381
Iwate Pref. * Year 2012	0.97 (0.92–1.03)	0.363	1.03 (0.91–1.15)	0.653	1.00 (0.90–1.13)	0.944	0.91 (0.81–1.02)	0.093	1.00 (0.84–1.20)	0.981
44 prefectures * Year 2013	Ref.		Ref.		Ref.		Ref.		Ref.	
Fukushima Pref. * Year 2013	0.95 (0.90–1.01)	0.091	1.03 (0.92–1.16)	0.565	1.02 (0.91–1.14)	0.759	1.01 (0.91–1.13)	0.823	0.79 (0.66–0.95)	0.011 ^†^
Miyagi Pref. * Year 2013	1.00 (0.94–1.06)	0.966	0.92 (0.82–1.04)	0.171	0.96 (0.86–1.08)	0.539	0.86 (0.77–0.97)	0.010 ^†^	0.90 (0.75–1.09)	0.283
Iwate Pref. * Year 2013	0.98 (0.93–1.04)	0.534	0.95 (0.85–1.07)	0.438	1.02 (0.91–1.14)	0.766	0.88 (0.78–0.98)	0.023 ^†^	1.12 (0.93–1.34)	0.233

RR, risk ratio; CI, confidence interval; Pref., Prefecture; *, interaction term; Ref, reference; ^†^ *p* < 0.05.

## Data Availability

Publicly available population data estimated by National Cancer Center Japan were analyzed in this study. These data can be found here: https://ganjoho.jp/reg_stat/statistics/dl/statistics_p05.html (accessed on 9 March 2023) (in Japanese). Meanwhile, restrictions apply to the availability of the death certificate data. These data were obtained from the Ministry of Health, Labour and Welfare of Japan and are available from https://www.mhlw.go.jp/toukei/sonota/chousahyo.html (accessed on 9 March 2023) (in Japanese) with permission.

## Appendix A

**Table A1:** Characteristics of the population data from 2006 to 2015.

		**Fukushima Pref.**		**Miyagi Pref.**		**Iwate Pref.**		**Others**	
		**Mean ^a^**	**%**	**Mean ^a^**	**%**	**Mean ^a^**	**%**	**Mean ^a^**	**%**
All		2007	100.0	2329	100.0	1316	100.0	2738	100.0
Age category									
0–64 years		1494	74.4	1797	77.1	951	72.3	2,091	76.4
≥ 65 years		513	25.6	532	22.9	365	27.7	647	23.6
65–69 years		125	6.2	140	6.0	88	6.7	182	6.6
70–74 years		115	5.7	125	5.4	85	6.4	157	5.7
75–79 years		108	5.4	110	4.7	79	6.0	129	4.7
80–84 years		87	4.3	84	3.6	61	4.6	94	3.4
85–89 years		52	2.6	48	2.1	35	2.6	54	2.0
90–94 years		21	1.0	19	0.8	14	1.1	23	0.8
≥95 years		6	0.3	6	0.3	4	0.3	8	0.3
								
Sex (age ≥ 65 years)									
Male		213	41.5	225	42.3	149	40.7	277	42.7
Female		300	58.5	307	57.7	217	59.3	371	57.3

^a^ Mean value was the mean annual population per 1000 calculated by dividing the sum of the population between 2006 and 2015 by 10 years. In addition, the mean values for the area category Others were divided by 44 prefectures. Pref.: Prefecture; Others: the 44 prefectures other than the Fukushima, Miyagi, and Iwate prefectures.

**Table A2:** Risk ratios and confidence intervals for fixed effects and interaction terms according to the cause of death.

	Neoplasm		Heart Disease		Stroke		Pneumonia		Senility	
Parameters	RR (95% CI)	*p*-Value	RR (95% CI)	*p*-Value	RR (95% CI)	*p*- Value	RR (95% CI)	*p*-Value	RR (95% CI)	*p*-Value
** Fixed effect**										
** Area**										
44 prefectures	Ref.		Ref.		Ref.		Ref.		Ref.	
Fukushima Pref.	1.00 (0.90–1.11)	0.979	1.17 (0.96–1.43)	0.128	1.20 (0.95–1.53)	0.134	0.96 (0.77–1.20)	0.731	1.35 (0.79–2.32)	0.278
Miyagi Pref.	1.01 (0.91–1.12)	0.882	0.99 (0.81–1.22)	0.956	1.16 (0.91–1.48)	0.221	0.89 (0.71–1.12)	0.319	1.26 (0.73–2.16)	0.407
Iwate Pref.	0.99 (0.89–1.10)	0.825	1.12 (0.92–1.37)	0.259	1.37 (1.07–1.74)	0.011 ^†^	1.00 (0.80–1.26)	0.965	1.06 (0.61–1.81)	0.846
										
** Year of death**										
2006	Ref.		Ref.		Ref.		Ref.		Ref.	
2007	0.99 (0.98–1.00)	0.055	0.97 (0.95–0.99)	0.010 ^†^	0.96 (0.94–0.98)	<0.001 ^††^	0.98 (0.96–1.00)	0.034 ^†^	1.03 (0.99-1.06)	0.152
2008	0.99 (0.98–1.00)	0.015 ^†^	0.98 (0.96–1.00)	0.048 ^†^	0.92 (0.90–0.94)	<0.001 ^††^	0.97 (0.95–0.99)	0.010 ^†^	1.12 (1.08-1.16)	<0.001 ^††^
2009	0.98 (0.97–0.99)	<0.001 ^††^	0.94 (0.92–0.96)	<0.001 ^††^	0.86 (0.84–0.88)	<0.001 ^††^	0.91 (0.89–0.93)	<0.001 ^††^	1.16 (1.12-1.20)	<0.001 ^††^
2010	0.99 (0.98–1.00)	0.047 ^†^	0.95 (0.93–0.97)	<0.001 ^††^	0.8 (0.81–0.85)	<0.001 ^††^	0.92 (0.90–0.94)	<0.001 ^††^	1.29 (1.24-1.33)	<0.001 ^††^
2011	0.97 (0.96–0.99)	<0.001 ^††^	0.94 (0.92–0.97)	<0.001 ^††^	0.80 (0.79–0.82)	<0.001 ^††^	0.92 (0.90–0.94)	<0.001 ^††^	1.43 (1.38-1.48)	<0.001 ^††^
2012	0.96 (0.95–0.97)	<0.001 ^††^	0.93 (0.91–0.95)	<0.001 ^††^	0.77 (0.75–0.79)	<0.001 ^††^	0.88 (0.86–0.90)	<0.001 ^††^	1.62 (1.56–1.68)	<0.001 ^††^
2013	0.95 (0.94–0.96)	<0.001 ^††^	0.87 (0.86–0.89)	<0.001 ^††^	0.72 (0.70–0.73)	<0.001 ^††^	0.85 (0.83–0.87)	<0.001 ^††^	1.78 (1.72–1.84)	<0.001 ^††^
2014	0.94 (0.93–0.95)	<0.001 ^††^	0.85 (0.83–0.87)	<0.001 ^††^	0.68 (0.66–0.69)	<0.001 ^††^	0.79 (0.77–0.81)	<0.001 ^††^	1.87 (1.80–1.93)	<0.001 ^††^
2015	0.94 (0.93–0.95)	<0.001 ^††^	0.83 (0.81–0.85)	<0.001 ^††^	0.65 (0.63–0.66)	<0.001 ^††^	0.78 (0.76–0.79)	<0.001 ^††^	2.05 (1.98–2.12)	<0.001 ^††^
										
** Age category**										
65–69 years	Ref.		Ref.		Ref.		Ref.			
70–74 years	1.47 (1.46–1.48)	<0.001	1.80 (1.76–1.83)	<0.001	1.84 (1.80–1.87)	<0.001	2.42 (2.38–2.46)	<0.001		
75–79 years	2.21 (2.19–2.23)	<0.001	3.53 (3.46–3.59)	<0.001	3.74 (3.68–3.81)	<0.001	6.11 (6.01–6.22)	<0.001	Ref.	
80–84 years	3.25 (3.22–3.28)	<0.001	7.34 (7.21–7.48)	<0.001	7.74 (7.60–7.88)	<.001	15.54 (15.27–15.82)	<0.001	5.18 (5.06–5.31)	<0.001
85–89 years	4.54 (4.50–4.58)	<0.001	15.04 (14.76–15.32)	<0.001	15.12 (14.85–15.40)	<0.001	36.61 (35.97–37.27)	<0.001	21.08 (20.57–21.60)	<0.001
90–94 years	5.71 (5.66–5.76)	<0.001	28.68 (28.15–29.21)	<0.001	26.73 (26.25–27.22)	<0.001	76.33 (74.99–77.70)	<0.001	79.20 (77.28–81.15)	<0.001
≥95 years	6.10 (6.05–6.15)	<0.001	50.05 (49.13–50.98)	<0.001	40.89 (40.16–41.64)	<0.001	139.85 (137.39–142.35)	<0.001	266.79 (260.35–273.39)	<0.001
										
Sex										
Male	Ref.		Ref.		Ref.		Ref.		Ref.	
Female	0.44 (0.44–0.44)	<0.001 ^††^	0.61 (0.60–0.61)	<0.001 ^††^	0.59 (0.59–0.60)	<0.001 ^††^	0.38 (0.37–0.38)	<0.001 ^††^	0.97 (0.95–0.98)	<0.001 ^††^
										
** Interaction terms**										
44 prefectures * Year 2010	Ref.		Ref.		Ref.		Ref.		Ref.	
Fukushima Pref. * Year 2010	1.01 (0.95–1.07)	0.730	0.99 (0.88–1.12)	0.895	1.04 (0.92–1.16)	0.546	1.03 (0.92–1.15)	0.589	0.89 (0.74–1.07)	0.217
Miyagi Pref. * Year 2010	1.01 (0.96–1.07)	0.643	0.97 (0.86–1.09)	0.624	1.08 (0.96–1.21)	0.179	0.93 (0.84–1.05)	0.238	1.16 (0.96–1.39)	0.117
Iwate Pref. * Year 2010	0.99 (0.93–1.05)	0.689	0.96 (0.85–1.08)	0.482	0.99 (0.88–1.11)	0.850	1.02 (0.91–1.14)	0.741	1.02 (0.85–1.23)	0.816
										
44 prefectures * Year 2011	Ref.		Ref.		Ref.		Ref.		Ref.	
Fukushima Pref. * Year 2011	0.98 (0.92–1.03)	0.435	1.06 (0.95–1.20)	0.302	1.08 (0.96–1.21)	0.200	1.10 (0.99–1.23)	0.086	0.98 (0.81–1.17)	0.789
Miyagi Pref. * Year 2011	0.97 (0.92–1.03)	0.334	1.03 (0.91–1.15)	0.674	1.13 (1.01–1.27)	0.031 ^†^	1.17 (1.04–1.31)	0.007 ^†^	1.28 (1.07–1.54)	0.007 ^†^
Iwate Pref. * Year 2011	0.99 (0.94–1.05)	0.719	1.06 (0.95–1.20)	0.294	1.07 (0.96–1.20)	0.224	1.04 (0.93–1.17)	0.480	0.97 (0.81–1.16)	0.733
										
44 prefectures * Year 2012	Ref.		Ref.		Ref.		Ref.		Ref.	
Fukushima Pref. * Year 2012	0.96 (0.91–1.01)	0.140	1.01 (0.90–1.14)	0.807	0.95 (0.84–1.06)	0.345	1.04 (0.93–1.16)	0.532	0.85 (0.71–1.02)	0.075
Miyagi Pref. * Year 2012	0.99 (0.94–1.05)	0.752	0.94 (0.84–1.06)	0.304	0.97 (0.86–1.09)	0.577	0.98 (0.87–1.09)	0.686	0.92 (0.77–1.11)	0.381
Iwate Pref. * Year 2012	0.97 (0.92–1.03)	0.363	1.03 (0.91–1.15)	0.653	1.00 (0.90–1.13)	0.944	0.91 (0.81–1.02)	0.093	1.00 (0.84–1.20)	0.981
										
44 prefectures * Year 2013	Ref.		Ref.		Ref.		Ref.		Ref.	
Fukushima Pref. * Year 2013	0.95 (0.90–1.01)	0.091	1.03 (0.92–1.16)	0.565	1.02 (0.91–1.14)	0.759	1.01 (0.91–1.13)	0.823	0.79 (0.66–0.95)	0.011 ^†^
Miyagi Pref. * Year 2013	1.00 (0.94–1.06)	0.966	0.92 (0.82–1.04)	0.171	0.96 (0.86–1.08)	0.539	0.86 (0.77–0.97)	0.010 ^†^	0.90 (0.75–1.09)	0.283
Iwate Pref. * Year 2013	0.98 (0.93–1.04)	0.534	0.95 (0.85–1.07)	0.438	1.02 (0.91–1.14)	0.766	0.88 (0.78–0.98)	0.023 ^†^	1.12 (0.93–1.34)	0.233

RR, risk ratio; CI, confidence interval; Pref., Prefecture; *, interaction term; Ref, reference; ^†^ *p* < 0.05; ^††^; *p* < 0.001.
